# Multi-cohort comparative analysis of salivary microbiotas reveals rural Ethiopians harbor a distinct composition correlated with lower esophageal cancer prevalence

**DOI:** 10.1128/msystems.00232-26

**Published:** 2026-06-02

**Authors:** Girma Mulisa, Jingcheng Zhao, Geda Lelissa, Iyunoluwa J. Ademola-Popoola, Anastacia Marie Diaz Huemme, Laura S. Weyrich, Adane Mihret, Tufa Gemechu, Abate Bane, Roger Pero-Gascon, Marthe De Boevre, Sarah De Saeger, Tamrat Abebe, Jordan E. Bisanz

**Affiliations:** 1 Department of Microbiology, Immunology and Parasitology, Addis Ababa University 37602, Addis Ababa, Ethiopia; 2 Department of Biomedical Sciences, Adama Hospital Medical College 247451, Adama, Ethiopia; 3 Department of Biochemistry and Molecular Biology, Pennsylvania State University 311285 , University Park, Pennsylvania, USA; 4 Department of Internal Medicine, St Paul's Hospital Millennium Medical College 443880, Addis Ababa, Ethiopia; 5 Department of Anthropology, Pennsylvania State University 311285 , University Park, Pennsylvania, USA; 6 One Health Microbiome Center, Huck Life Sciences Institute 124474, University Park, Pennsylvania, USA; 7 Department of Bioethics, Rock Ethics Institute, Pennsylvania State University 8082, University Park, Pennsylvania, USA; 8 School of Biological Sciences, Adelaide University 1066 , Adelaide, South Australia, Australia; 9 Armauer Hansen Research Institute, Ministry of Health 70605, Addis Ababa, Ethiopia; 10 Department of Pathology, School of Medicine College of Health Sciences, Addis Ababa University 37602, Addis Ababa, Ethiopia; 11 Department of Internal Medicine, School of Medicine College of Health Sciences, Addis Ababa University 37602, Addis Ababa, Ethiopia; 12 Center of Excellence in Mycotoxicology and Public Health, Faculty of Pharmaceutical Sciences, Ghent University 26656, Ghent, Belgium; 13 Department of Biotechnology and Food Technology, Faculty of Sciences, University of Johannesburg 61799, Johannesburg, South Africa; 14 Center of Molecular Toxicology and Carcinogenesis, Pennsylvania State University 8082, University Park, Pennsylvania, USA; North Carolina Agricultural and Technical State University, Greensboro, North Carolina, USA

**Keywords:** esophageal cancer, saliva, microbiome, meta-analysis

## Abstract

Esophageal cancer (EC) results in high mortality due to difficulty in early diagnosis, particularly in low- and middle-income countries, including the African EC belt typified by high prevalence, early onset, and poor prognosis. While the precise etiological factors remain unknown, emerging data suggest links to the oral microbiota. In this study, we conducted a secondary analysis using V4 16S rRNA sequencing from a cross-sectional study of treatment-naive, newly diagnosed EC patients (*N* = 103) and healthy controls (*N* = 108) residing in agricultural regions of Ethiopia. We report that the salivary microbiota in the healthy Ethiopian controls is highly diverse, forming two functionally distinct community clusters differing in diversity, composition, and absolute abundance. Microbiota composition was associated with sex and alcohol consumption, but not age. Comparisons against groups from geographically distinct populations representing Tanzania, Uganda, Venezuela, and the United States (*N* = 641) showed that cluster 2 resembled other East African populations, while cluster 1 was unique to the Ethiopian cohort. Both EC subtypes, esophageal squamous cell carcinoma (ESCC) and esophageal adenocarcinoma, were associated with a loss of microbial diversity and an increased probability of having a cluster 2 microbiota (adjusted OR = 2.9 [95% CI 1.5–5.9]). Classifiers trained to discriminate healthy and EC samples were further validated on two external EC cohorts from China (*N* = 161). Models trained on the Ethiopian cohort could predict disease status in an external cohort of mid- and late-stage ESCC from China (AUROC = 0.70 ± 0.03 [mean ± SD]), demonstrating the generalization of microbial features of ESCC across populations.

**IMPORTANCE:**

Recent reports in North America and China have correlated oral microbiota composition with esophageal cancer, although the translation of this knowledge into the African esophageal cancer belt is hampered by a lack of data on the oral microbiota of East Africans and limited cross-cohort comparative analyses validating the utility of these biomarkers. We report that the human salivary microbiota is a meaningful biomarker of later-stage esophageal cancer that transcends geography and ethnicity and may provide utility for large-population screening. A lower-diversity and lower-abundance salivary microbiota correlated with esophageal cancer warrants further investigation to understand the role of oral microbes in mediating carcinogenesis.

## INTRODUCTION

Cancer is the second leading cause of death worldwide, and it is estimated that one in five individuals will develop cancer in their lifetime, with 1 in 9 men and 1 in 12 women dying from cancer (1). This proportion is higher in low- and middle-income countries (LMICs) (2), including Ethiopia, where cancer-related mortality and incidence rates have increased in the last two decades (3). Esophageal cancer (EC) consists of two main subtypes: esophageal squamous cell carcinoma (ESCC) and esophageal adenocarcinoma (EAC) (4). ESCC accounts for approximately 90% of EC worldwide and is more common in LMICs, while EAC is more common in developed countries and is increasing globally (5, 6). Prominent geographic variation in the incidence of EC has been observed, signifying the role of local environmental factors in disease occurrence. Eastern and Southern Africa and Eastern and South Central Asia are regions where the incidence of EC and associated mortality is disproportionately high (7) while also exhibiting higher rates of ESCC compared to Western countries (8–10).

Ethiopia is among the countries of the African EC belt (11), with EC among the top 10 cancer types (12). The African EC belt extends along the eastern countries of Africa, including Ethiopia, Kenya, Tanzania, Malawi, Mozambique, and South Africa, exhibiting geographic and ethnic variation at the local level. Furthermore, there is a concerning trend of significant increases in incidence (11). EC is particularly high in the Arsi-Bale districts of Oromia regional state in Ethiopia, accounting for over 50% of Ethiopian cases (13–15). In total, 80% of EC patients in Ethiopia present advanced stages of the disease (stages III and IV) (14), with a median survival time after diagnosis of 4 months (16). Endoscopy is the only validated technique for the screening and diagnosis of EC, but it involves invasive techniques and requires specialized training and resources not widely available in resource-limited settings, including rural Ethiopia. There is no approved alternative non-invasive method for the diagnosis and screening of EC. Taken together, these data demonstrate the urgent need to identify the risk factors and search for alternative diagnostic methods.

Distinct risk factors have been identified for the two major histologies of EC: EAC and ESCC (17). Gastroesophageal reflux disease, obesity, and cigarette smoking are well-established risk factors of EAC (18, 19). In Western countries, cigarette and alcohol usage are the main established risk factors of ESCC (8); however, in LMICs, alternative risk factors have been suggested, including micronutrient deficiencies, low diet quality, consumption of betel/areca drinks, consumption of high-temperature foods/drinks, and an individual’s exposome (e.g., mycotoxins and polycyclic hydrocarbons) (8, 10, 20). Low socio-economic status and poor oral health have been further identified. In Ethiopia, ESCC is the predominant histological type, and established risk factors in Western populations, such as alcohol and tobacco use, are low in individuals with EC (21, 22). Few descriptive studies have identified the epidemiological, behavioral, nutritional, and environmental factors of EC in Ethiopia (14, 21–23). In the absence of strong predictive risk factors, additional studies are needed in this region.

There is growing evidence that human-associated microbes, the microbiota, play a role in the occurrence, pathogenesis, and treatment outcomes of many cancer types (24–26). While much research has focused on the gut microbiota (27), the oral microbiota is associated with an increased risk of pancreatic (28), colorectal (29), and lung cancers (30). Connections between esophageal cancer and the oral microbiota have been previously explored in the United States and China. A prospective study in the United States reported higher richness and abundances of *Tannerella forsythia*, *Streptococcus pneumoniae*, *Neisseria* spp., and *Porphyromonas gingivalis* as predictors of EC (25). Alternatively, a Chinese study showed that ESCC cases have an overall decreased microbial diversity compared to healthy controls and dysplasia subjects (24). Combined epidemiological risk factors and oral microbiome biomarkers have been proposed to be used as non-invasive early screening markers of ESCC in China, although similar studies in other countries are needed to increase the global validity of the method (26).

Oral microbiota research, similar to other body sites, has been primarily focused on industrialized societies in the global West (31), termed Western populations, although we know that oral microbiotas are distinct across discrete human populations (32) and shaped by one’s diet, environment, genetics, hygiene, and lifestyle (33–35). As such, it is plausible that unique oral microbes in understudied populations play yet-undescribed roles in disease or that non-Western microbes can aid in identifying population-specific disease biomarkers (36–38). These observations strongly demonstrate the need to diversify study populations and conduct medically relevant studies in underrepresented populations, especially in the African continent (39). Understanding and accurately classifying similarities and differences between Western, westernized populations adopting similar dietary and lifestyle practices, and non-westernized populations may allow for more accurate biomarker identification and provide a more comprehensive global picture of oral microbiome diversity. Comparative oral microbiota studies across global populations will help uncover variation in disease etiologies and susceptibilities, novel adaptive mechanisms, and potential prophylactic strategies.

In this report, we sought to test the hypothesis that the salivary microbiota is a biomarker of esophageal cancer across populations. As such, we characterized the salivary microbiota of EC patients and controls to characterize the oral microbiota of healthy rural Ethiopians and to uncover potential biomarkers for EC. We find that the healthy oral microbiota of Ethiopians is highly diverse and forms two functionally distinct populations. Cross-sectional analysis of EC and controls reveals the loss of microbial diversity and an altered community composition associated with EC. We further performed cross-cohort analyses, identifying a unique subpopulation distinct from other East African cohorts and demonstrating that EC-associated microbial patterns can be translated to Chinese cohorts. These data extend our understanding of the oral microbiota in non-Western/westernized populations and reveal translational targets for diagnosis and prevention of EC in global populations.

## RESULTS

### Demographic characteristics of the study cohort

Salivary microbiota from a total of 108 non-cancerous healthy controls and 103 newly diagnosed treatment-naive EC participants were successfully sequenced. Relevant participant demographics are displayed in Table 1. All the EC participants and controls were rural residents of Arsi, Bale, East Shoa, and West Harage districts of the Oromia region in Ethiopia. ESCC was the most common histological type, accounting for 85% of EC participants (87/103 individuals). EC participants were, on average, 12.5 years older than controls (*P* = 2.3e−14, Mann-Whitney *U* test). Compared to controls, there were significantly more men in the EC group, representing 26% and 40% of the control and EC groups, respectively (*P* = 0.028, Fisher’s exact test). Usage of cigarettes, chewing tobacco, and khat was reported by fewer than five individuals per group and found to not be significantly different (Table 1). Alcohol usage was reported by 12% of participants and was significantly lower in the EC participants (*P* = 0.006, Fisher’s exact test). All participants reported occupations associated with farming/agriculture. Coffee consumption was reported by 95.7% of participants, which was marginally higher in EC participants (*P* = 0.003, Fisher’s exact test).

**TABLE 1:** Participant demographics

Variable (units)	Healthy controls (*N* = 108)	EC participants (*N* = 103)	*P*-value^*a*^
Age (mean ± SD)	39.0 ± 7.1	51.5 ± 13.8	2.3e−14
Sex (M/F)	28/80	42/61	0.028
Cigarette use (N)	0	3	0.11
Chewing tobacco use (*N*)	0	3	0.11
Alcohol use (*N*)	20	6	0.0060
Khat use (*N*)	4	5	0.74
Coffee use (*N*)	99	103	0.0033

^*a*^ Mann Whitney *U* test for age and Fisher’s exact test for categorical variables.

### Rural Ethiopian oral microbiotas are highly diverse and form two distinct clusters

**Fig 1:**
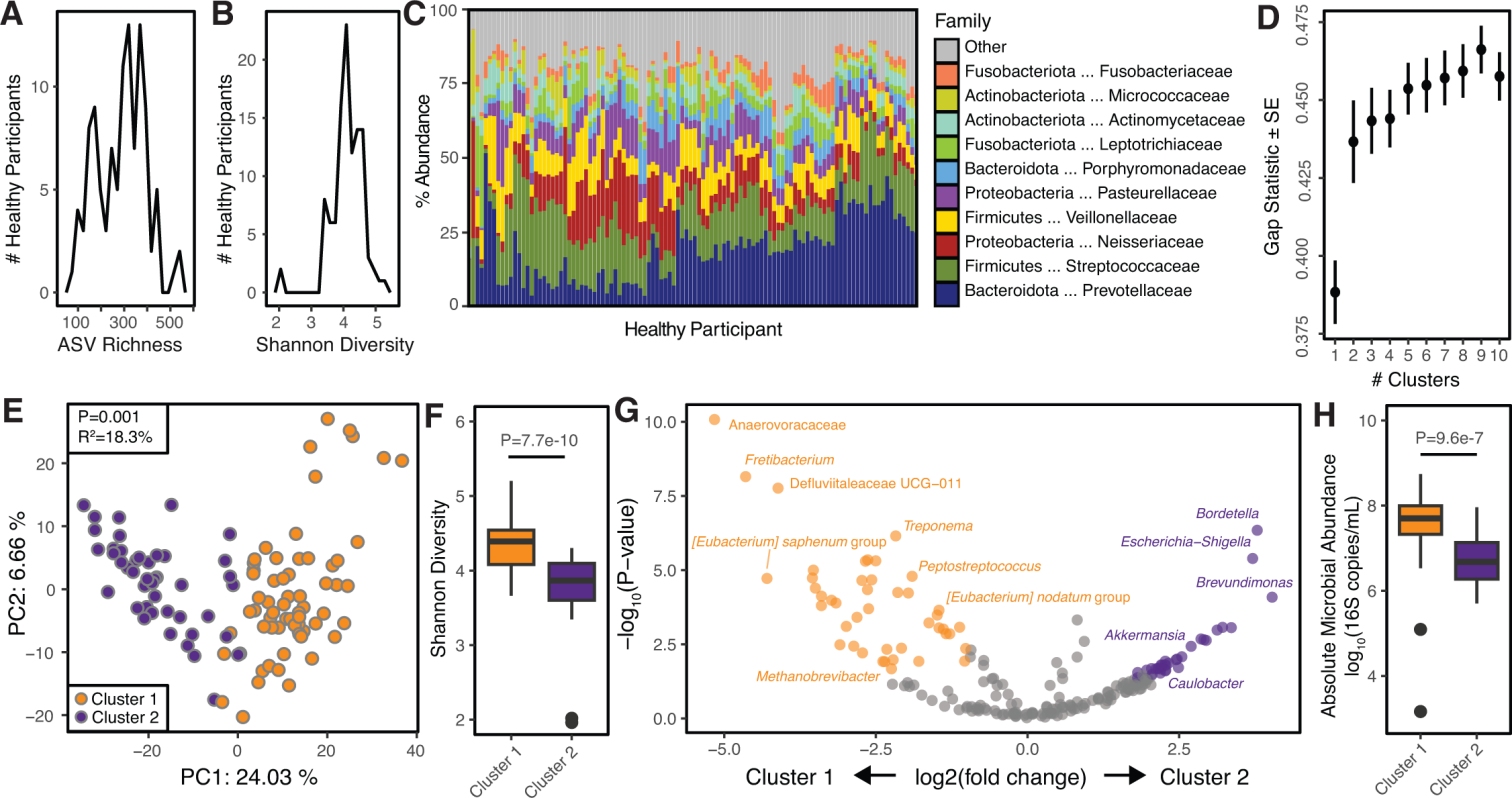
Two diverse and compositionally distinct oral microbiota types are observed among healthy rural Ethiopians. Diversity of oral microbiotas in healthy controls measured by (**A**) the richness of amplicon sequence variants (ASVs) and (**B**) Shannon’s diversity index. (**C**) A barplot of family-level abundances illustrates that the most commonly observed oral microbes belong to the Prevotellaceae and Streptococcaceae. (**D**) Partitioning around medoids (PAM) clustering, with analysis of the gap statistic, demonstrates that two clusters best describe the number of community types. (**E**) PCoA analysis of Aitchison distance based on genus-level abundances between healthy participants demonstrates two coherent clusters with community state type describing 18.3% of variation (*P* = 0.001, PERMANOVA). (**F**) Microbial diversity is significantly lower in cluster 2 (Welch’s *t*-test). (**G**) Significantly different genera between community clusters (ALDEx2 FDR-corrected Welch *t*-test < 0.1). (**H**) The microbial load is significantly lower in cluster 2 as determined through qPCR (Welch’s *t*-test). *N* = 108 healthy participants in panels A–H. *N*_Cluster1_ = 64, *N*_Cluster2_ = 44.

A total of 3,533 denoised amplicon sequence variants (ASVs) were detected across the healthy control cohort (*N* = 108). The median number of ASVs on a per-individual basis was 302, ranging from 79 to 546 (Fig. 1A). Richness of ASVs was high and also evenly distributed within individuals (Fig. 1B). The most abundant oral microbes belonged to the Prevotellaceae, Streptococcaceae, and Neisseriaceae families (Fig. 1C; Fig. S1A). In both the analysis of taxonomic composition and through beta-diversity analysis, a qualitative pattern emerged, suggesting the presence of discrete compositional clusters. An unsupervised partitioning around medoids (PAM) clustering approach was applied, which provided evidence of two compositional clusters as determined through maximizing the gap statistic in the minimal number of clusters (Fig. 1D). Ordination of samples provided visual confirmation that these clusters mapped to an apparent bifurcation in community composition (Fig. 1E), which was consistent with our previous results identifying two distinct oral communities in pre-industrialized European populations (40). This clustering was robust to the strict removal of any putative contaminant ASV observed in either negative or positive controls (see Materials and Methods). Notably, community clusters had no correspondence with DNA extraction plate, physical locations in multi-well plates, sex, age, or other demographic variables. Cluster 2 microbiotas exhibited lower microbial diversity (Fig. 1F).

Differential abundance analysis between clusters revealed 73 significantly different genera, 43 associated with cluster 1, and 30 with cluster 2 (FDR < 0.1, ALDEx2; Fig. 1G). Cluster 1 was associated with a broad spectrum of bacterial genera previously described in the oral cavity but notably included the archaeal genus *Methanobrevibacter*. Alternatively, cluster 2 was associated with a number of Proteobacteria genera, including *Bordetella*, *Brevundimonas*, and *Escherichia*, as well as the common gut genus *Akkermansia*. We quantified the absolute abundance of microbes and discovered a reduction in the microbial load (difference in log_10_ means = 0.8 [0.5–1.1 95% CI]; Fig. 1H). Through metagenomic inference (PICRUSt; Fig. S1B), cluster 1 was associated with archaeal pathways involved in methanogenesis, while cluster 2 was associated with a number of pathways, including those involved in amino acid metabolism. Taken together, these findings indicated a potential population structure within the healthy cohort defined by the composition, function, and absolute abundance of salivary microbes.

**Fig 2:**
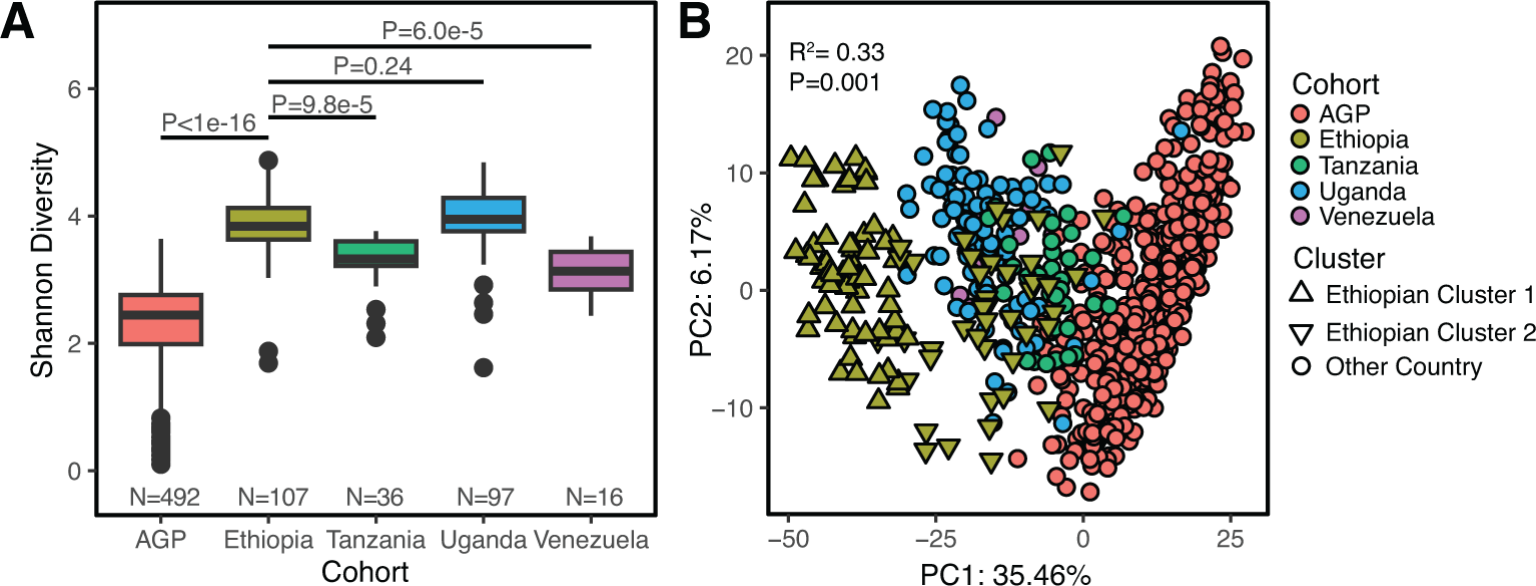
Cluster 1 healthy rural Ethiopian oral microbiotas are distinct from other non-Western populations. (**A**) Rural Ethiopian oral microbiotas display higher diversity than westernized (AGP) and Tanzanian communities but are not significantly more diverse than those from Uganda (ANOVA with Tukey HSD). (**B**) PCoA analysis of Aitchison distances displays distinct compositional differences between westernized (AGP) and non-westernized communities (inset statistical analysis by PERMANOVA). While cluster 2 Ethiopian microbiota are not distinct from other East African populations, cluster 1 communities are distinct.

To understand if this bifurcation in community structure was unique to the rural Ethiopian cohort, the composition of the healthy Ethiopian controls was contrasted against oral microbiota profiles previously collected from other noncancerous East African cohorts in Uganda, and those we previously characterized in Tanzania (41). These were further contrasted against a non-westernized population from Venezuela (42) and samples from the American Gut Project (AGP) (43). Samples from the AGP are primarily from individuals in the United States; however, it also contains samples from other westernized populations (i.e., mostly the United Kingdom and Australia). A comparison of methodological variables between studies is included in Table S2. All samples in this analysis were surveyed using V4 16S rRNA sequencing on Illumina platforms with 515F/806R primers. All utilized mechanical disruption during extraction. These methodological considerations are important, as recent studies have demonstrated that they explain the largest sources of variation across global cohorts (44). Oral microbiota diversity was significantly higher in all non-westernized populations compared to the AGP cohort (*P* < 2.9E−6 ANOVA with Tukey HSD; Fig. 2A). The Ethiopian and Ugandan cohorts displayed significantly higher diversity than the Tanzanian and Venezuelan cohorts but were not significantly different from each other. Analysis of taxonomic abundances revealed notable differences among the Ethiopian cohort and all other reference cohorts (Fig. S2). Among the notable differences contrasting the healthy Ethiopian cohort against the AGP were higher levels of Prevotellaceae and lower levels of Enterobacteriaceae, Micrococcaceae, and Pseudomonadaceae (FDR < 0.1, ALDEx2). When beta-diversity analysis was performed (Fig. 2B), the samples formed a gradient along PC1, drawing a clear delineation between Western and non-westernized populations (effect of cohort: *R*^2^ = 0.33, *P* = 0.001 PERMANOVA). Interestingly, the previous population structure of the two Ethiopian clusters was retained when ordinated with the reference cohorts, demonstrating that Ethiopian cluster 2 was compositionally similar to other East African cohorts, but Ethiopian cluster 1 was distinct.

**Fig 3:**
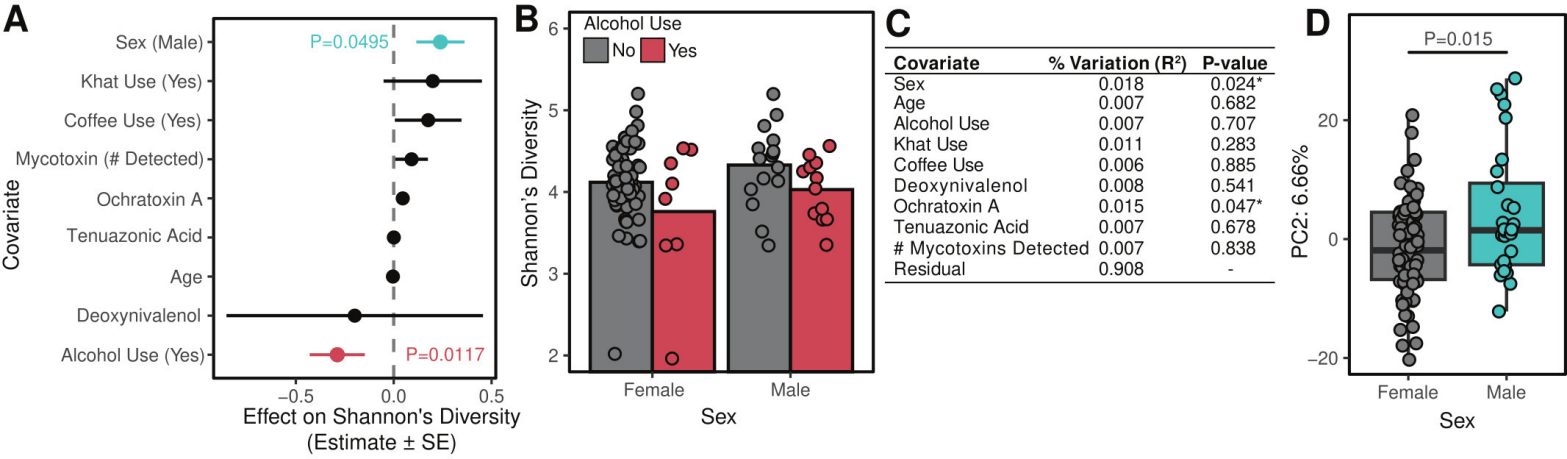
Oral microbiota composition is impacted by sex and alcohol consumption. (**A**) Oral microbiota diversity is significantly associated with sex and alcohol consumption (*P* < 0.05, generalized linear model, *N* = 108, AIC = 153.84). (**B**) Identifying as male is associated with higher microbial diversity, while both sexes show a decrease in diversity associated with reported alcohol consumption. (**C**) Analysis of microbial community composition demonstrates that sex and serum ochratoxin A are associated with altered community composition (*P* < 0.05, PERMANOVA of CLR Euclidean distances). (**D**) Sex is associated with the second axis of the principal coordinates plot shown in Fig. 1E rather than the community clusters associated with PC1 (Welch’s *t*-test).

To better understand the factors that may shape oral microbiota composition in the healthy Ethiopian cohort, we examined relevant covariates that determined community diversity and composition. Given that mycotoxin exposure may be etiologically linked to EC, we included additional covariates of plasma mycotoxin levels, which we have previously described (45). Notably, mycotoxin burden differs significantly between EC participants and controls, although both groups have significant exposure (Table S3). The final generalized linear model, which best fit the data, found significant effects of sex and alcohol use, but not age, on alpha diversity (Fig. 3A). Coffee consumption and khat usage, as well as the number of mycotoxins detected in plasma, and the plasma concentration of ochratoxin A, were found to approach the significance threshold. Reporting as male was associated with a higher Shannon diversity index (0.227 ± 0.11, *P* = 0.0495), while alcohol consumption was associated with a lower alpha diversity (−0.330 ± 0.129, *P* = 0.0117; Fig. 3B). In a similar analysis of community composition/beta diversity, significant effects of sex (*R*^2^ = 0.018, *P* = 0.024, PERMANOVA) and plasma ochratoxin A (*R*^2^ = 0.015, *P* = 0.047) were observed (Fig. 3C). None of these variables appear to be predictive of the gradient along PC1, which defined the community clusters (Fig. 1C), although sex appeared to be represented on the second axis of the PCoA plot (Fig. 3D).

### The oral microbiota is disrupted in EC participants compared to healthy controls

**Fig 4:**
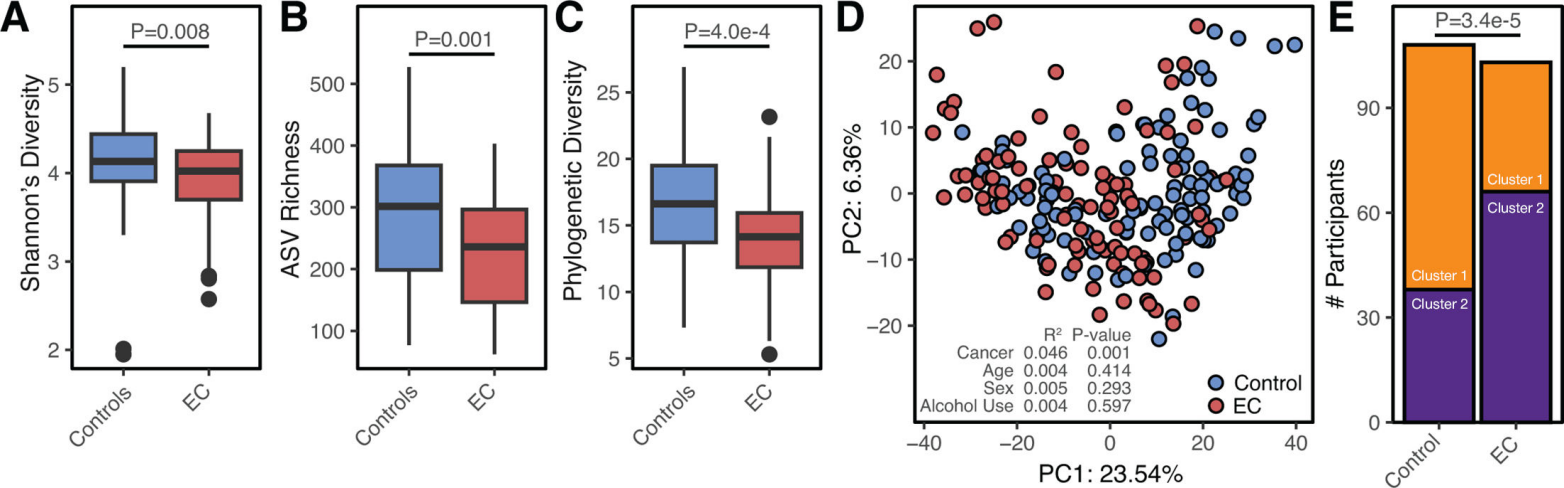
Oral cancer is associated with reduced diversity and community cluster 2. Microbial diversity is significantly reduced, irrespective of the choice of alpha diversity metric, including (**A**) Shannon’s diversity index, (**B**) ASV richness, and (**C**) phylogenetic diversity. (**D**) Visualization by PCoA demonstrates subtle variation in microbiome composition related to cancer status, which is supported by statistical analysis (inset, PERMANOVA). (**E**) Clustering analysis, as in Fig. 1, demonstrates EC participants are enriched in community cluster 2 microbiotas (OR = 3.3 95%CI 1.8–6.0; Fisher’s exact test). Statistical analysis for panels **A** and **C** by GLM with covariates of sex, age, and alcohol use. Statistical analysis for panel B was performed using the same with the negative binomial distribution. *N*_EC_ = 103, *N*_controls_ = 108 for panels **A–E**.

Cross-sectional analysis of microbial diversity measured through multiple alpha diversity metrics and accounting for relevant covariates revealed significant reductions in microbial diversity in EC participants (Fig. 4A through C). Analysis of community composition by ordination demonstrated clustering by disease status, which was found to be statistically significant (*R*^2^ = 0.046, *P* = 0.001 PERMANOVA; Fig. 4D). Subgroup analysis within the EC participants, contrasting EAC and ESCC subtypes, did not find significant differences in alpha or beta diversity (Fig. S3). Noting a trend of separation between EC participants and controls along the PC1 axis, the full set of samples (*N* = 211) was re-clustered as before using unsupervised methods that further supported the notion of the two community composition clusters previously observed, with a high correspondence to the previous assignments observed in the healthy cohort alone (Fig. 1D and E and 4D). Analysis of the distribution between community clusters revealed that community cluster 2 was enriched in the EC participants (OR = 3.3, 95%CI 1.8–6.0, Fisher’s exact test). This was further supported in a multivariate logistic regression adjusting for age, sex, and alcohol consumption (adjusted OR = 2.9 [95% CI 1.5–5.9]; Table 2). While cluster 2 was more common in EC participants, it was still prevalent in the control group (Fig. 4E), which may suggest potential value as a predictive tool in assessing disease risk.

**TABLE 2:** Logistic regression crude and adjusted odds ratios for predictors of esophageal cancer

Variable	Crude (univariate)		Adjusted (multivariate)	
	OR (95% CI)	*P*-value	OR (95% CI)	*P*-value
Cluster 2 (vs. 1)	3.3 (1.9–5.8)	3.6e−5	2.9 (1.4–5.9)	0.0025
Age (per year)	1.1 (1.1–1.2)	2.2e−10	1.2 (1.1–1.2)	1.9e−9
Male (vs. female)	2.0 (1.1–3.6)	0.023	1.2 (0.5–2.8)	0.64
Alcohol consumption	0.3 (0.1–0.7)	0.0077	0.07 (0.02–0.25)	6.8e−5

**Fig 5:**
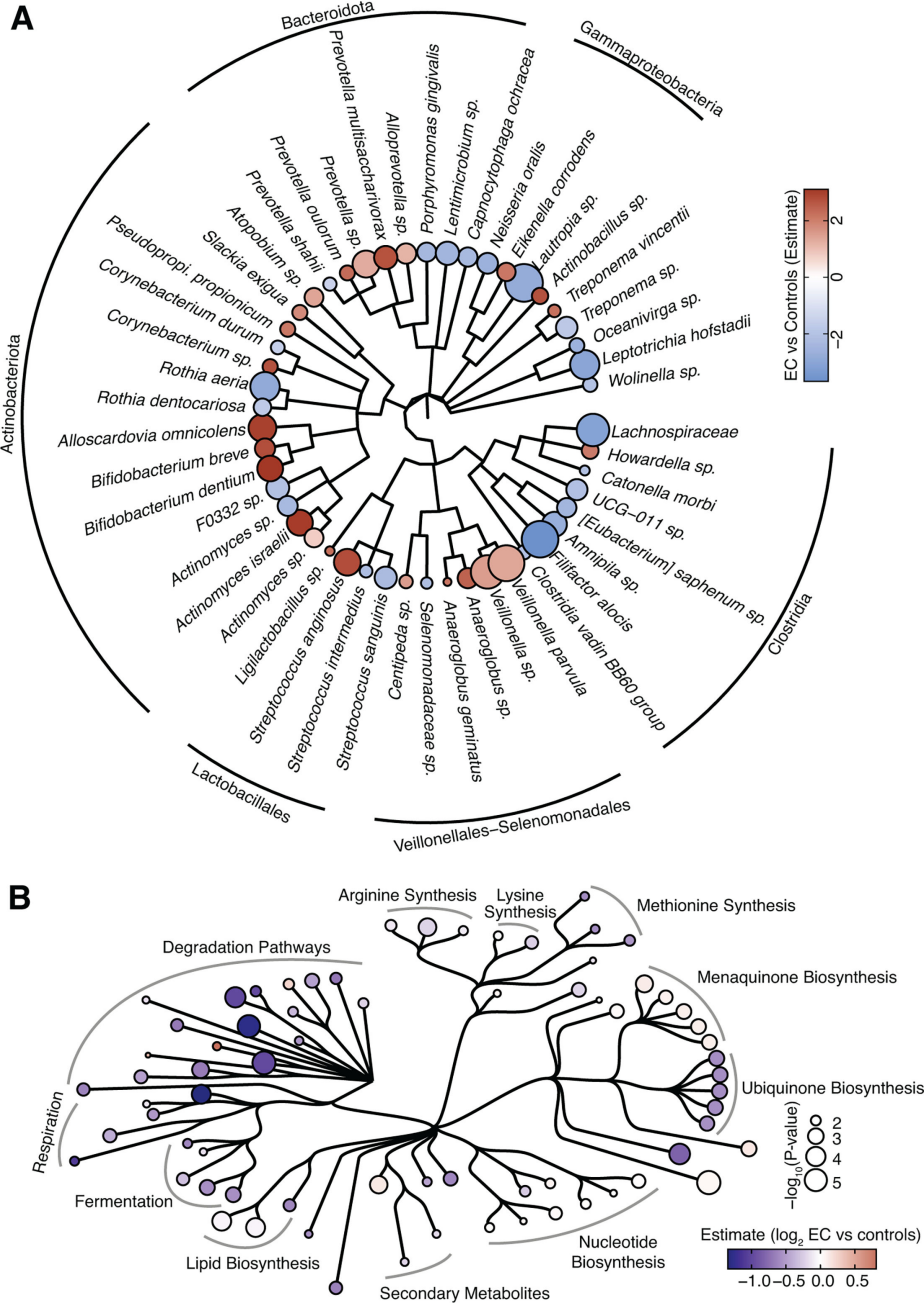
Esophageal cancer is associated with a functionally distinct microbiota. (**A**) Prokaryotic species differentially abundant between EC participants and controls are displayed in a cladogram based on taxonomy. (**B**) Inferred genetic pathways differentially abundant between EC participants and healthy controls are drawn in a functional hierarchy, highlighting ubiquinone/menaquinone biosynthesis, fermentation, and other degradative pathways associated with EC-related dysbiosis. Statistical analysis for panels **A** and **B** by GLM with covariates of sex, age, and alcohol use. Significance was determined as an FDR-corrected *P*-value < 0.1. No significant effects were observed for covariates. *N*_EC_ = 103, *N*_controls_ = 108 for all panels.

A cross-sectional analysis between EC participants and controls reported 49 differentially abundant species, 24 elevated in EC, and 25 elevated in controls (ALDEx2 GLM FDR < 0.1, Table S4). These organisms represented a broad phylogenetic range of common host-associated microbes with closely related organisms differing in their association (Fig. 5A). As representatives, *Streptococcus sanguis* and *S. intermedius* were negatively associated with EC, while *S. anginosus* was positively associated. Similarly, *Prevotella shahii* was associated with controls, while four other clades of *Prevotella/Alloprevotella* were associated with EC. Interestingly, the most enriched taxa in EC participants belonged to the Actinobacteria, representing *Bifidobacterium* spp., *Actinomyces,* and *Alloscardovia* (Table S4). Functional inference revealed that the most strongly enriched predicted pathway in EC participants was related to glycerol degradation to 1,3-propanediol (Table S5). There is an apparent bifurcation in quinone biosynthesis wherein controls indicate higher inferred abundance of pathways involved in ubiquinone biosynthesis, while EC participants display higher inferred abundance of those involved in the biosynthesis of menaquinone (Fig. 5B). EC was further associated with decreased predicted pathway abundances involved in the biosynthesis of amino acids and nucleotides and multiple pathways involved in fermentation, respiration, and degradation (Fig. 5B; Table S5).

### Esophageal cancer-associated microbiota signatures generalize across geographical cohorts

To better understand the reproducibility of the EC-associated shifts in the oral microbiota and if these may generalize to other populations, we sought to use a meta-analysis framework to conduct cross-population analysis. Of previous studies characterizing the oral microbiota using comparable sequencing methodologies, we were only able to obtain publicly available data from two studies (46, 47), both from Chinese populations that exhibit increased rates of ESCC. Comparisons of methodological variables are provided in Table S2. The cohort reported by Chen et al. consisted of individuals with early-stage ESCC (*N* = 31) and controls (*N* = 21) located around Nanjing. Alternatively, the Jiang et al. cohort consisted of 109 individuals recruited from Huaian, with either biopsy-diagnosed ESCC (*N* = 56) or healthy controls (*N* = 53). These locations are approximately 140 km apart and are found in eastern China. Taxonomic comparison across cohorts revealed similar compositions at the family level; however, the Chen et al. cohort exhibited lower levels of oral Prevotellaceae, while the Jiang et al. cohort exhibited lower levels of Streptococcaceae (Fig. S4A). Ordination of beta diversity metrics revealed that the Chinese cohorts most closely resembled each other, compared to the Ethiopian cohort (Fig. S4B). Cohort explained the major source of variation in the combined data set (*R*^2^ = 0.161, *P* = 0.001), while the effect of disease state was still statistically significant (*R*^2^ = 0.019, *P* = 0.001, PERMANOVA). Notably, differences in microbial alpha diversity were not observed in the Chinese cohorts (Fig. S4C).

**Fig 6:**
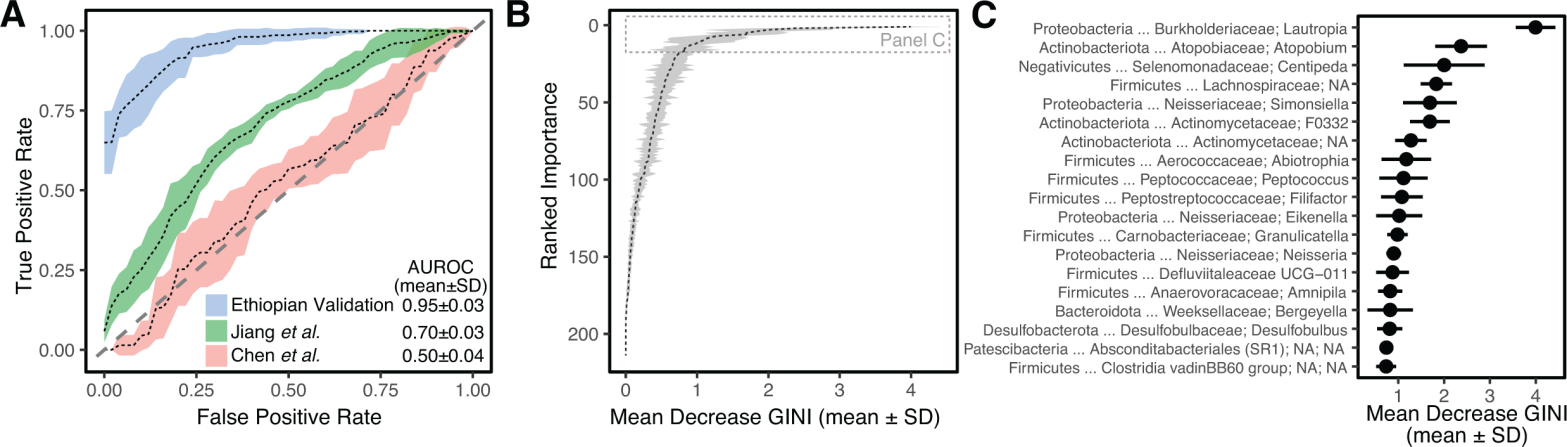
Shifts in the Ethiopian cohort predict disease status in a Chinese ESCC cohort, but not in an early-stage ESCC cohort. (**A**) Random forest classifier models were trained on 100 iterations of random sampling of the Ethiopian cohort into training (*N* = 139) and validation sets (*N* = 72; see Materials and Methods). Models were then used to predict cancer status in Chinese cohorts with good predictive power in the Jiang et al. cohort but not the Chen et al. cohort (see inset table of AUROCs). Ribbons represent mean ± SD of 100 replications. (**B**) A limited set of bacterial genera predicts cancer status (*N* = 20). as displayed in panel **C**.

To better understand how finer-scale changes in microbiota generalize across cohorts and to identify the most important predictors of cancer status, we used a robust machine learning approach consisting of 100 iterations of randomly sampling the Ethiopian cohort into training and test sets (Monte Carlo cross-validation), followed by the use of random forest classifiers generated from the training set to predict both the test set and external Chinese cohorts (see Materials and Methods). Models trained within the Ethiopian cohort had excellent accuracy in predicting the withheld Ethiopian samples, reporting areas under the receiver operator curve (AUROCs) of 0.95 ± 0.03 (mean ± SD; Fig. 6A). These models also gave good performance on the Jiang et al. cohort (AUROC = 0.70 ± 0.03), but not the Chen et al. cohort (AUROC = 0.50 ± 0.04). These observations likely reflect a biological difference between early-stage (Chen) and later-stage EC (Jiang); however, they may also result from technical difficulties in direct comparison of studies employing differing methodologies for sample collection and processing (48) (Table S2). Examination of the predictive power of individual taxa revealed an inflection point wherein approximately 20 genera most effectively differentiated EC participants and controls, of which *Lautropia* spp. was the strongest predictor (Fig. 6B and C). Taken together, these results indicate that while the Ethiopian and Chinese cohorts have distinct oral microbiotas, there is a common signature of mid- and late-stage disease observed across populations that suffer from high EC/ESCC burden.

## DISCUSSION

In this report, we characterized the salivary microbiota of healthy individuals residing in the rural region of Oromia, Ethiopia, and contrasted these profiles against other East African, Asian, South American, and westernized populations, uncovering a unique population structure found within Ethiopians. Contrasting healthy controls against individuals with esophageal cancer, we uncovered a loss of microbial diversity and an altered microbial composition associated with cancer: both the ESCC variant common to Africa, and the EAC variant more common in the global west. Through machine learning approaches, we demonstrated that taxonomic shifts in microbial community composition could predict current cancer status in an external Chinese cohort, highlighting a minimal number of strong predictors of disease status. Collectively, our study demonstrates the importance of studying non-westernized populations and reinforces a role for the oral microbiota as a biomarker and/or mediator of esophageal cancer.

While evidence has been provided of discrete community configurations in the gut and urogenital tract, commonly termed enterotypes (49) and community state types (50), respectively, this conceptual framework has been less frequently applied to the oral cavity. One study of adolescents in Spain reported two clusters, which they termed stomatotypes, although the taxa that define these clusters differ substantially from those we report (51). While these clustering approaches have drawn criticism (52), they remain useful tools for dimensional reduction and interpretation of highly complex data (53). Mimicking what has been described with gut enterotypes (54), we report that the community clusters observed in the healthy Ethiopian population differ in absolute abundance by nearly an order of magnitude. Similar clustering is observed in pre-industrialized Europeans, which is demarcated by the presence of either oral *Methanobrevibacter* or *Streptococcus* spp. (40), which are broadly similar to the Ethiopian cluster 1 and cluster 2, respectively. This fits with our observations that Ethiopian cluster 2 more is compositionally similar to the AGP cohort. Comparison to international cohorts from East Africa (Tanzania and Uganda), South America (Venezuela), and a predominantly American cohort (American Gut Project) also revealed that non-westernized cohorts exhibit significantly higher microbial diversity and higher levels of the family representing *Prevotella* spp., organisms known to be significantly higher in the fecal microbiota of African individuals (55). The observation that Ethiopian cluster 2 individuals from rural environments are more compositionally similar to westernized populations and that *Methanobrevibacter*-associated oral communities are absent in Western populations (40) may be indicative of signatures of a westernization process taking place within this unique Ethiopian cohort. The specific behavioral, dietary, or social/cultural factors driving this process remain unclear, although increased dietary carbohydrate consumption and altered dental hygiene practices have been suggested. In pre-industrial Europeans, the *Streptococcus*-associated oral community, but not the *Methanobrevibacter*-associated community, was linked with poorer oral health (40). Strikingly, Ethiopian cluster 2 was associated with EC in this study, which may suggest a hypothesis that loss of ancestral protective, non-industrialized microbes in the saliva may predispose an individual to EC; however, further studies are required to support this notion.

Irrespective of unsupervised clustering, we observed the diversity of salivary microbes to be reduced in EC individuals, which has been variably reported across previous studies (24, 56–58). Reduced microbial diversity may be associated with latent infection and inflammation (59). While it is difficult to establish cause and effect, the esophagus is prone to the disruption of barrier function due to both external factors, for example, hot food/beverages and dietary carcinogens, and due to its proximity to the low pH content of the stomach. These factors may interact with both resident and transitory microbes from the oral cavity to exacerbate inflammatory processes leading to carcinogenesis (60). Supporting these observations, gastroesophageal reflux disease (GERD), a known risk factor for EAC, is also associated with disrupted microbial communities, including elevated abundance of gram-negative microbes (61).

Some of the species we correlated with EC have been previously associated with various malignancies, including esophageal cancer. *S. anginosus* has been experimentally demonstrated to promote gastric tumorigenesis via inducing gastritis, which has been proposed as a potential biomarker for gastric cancer (62). *Prevotella* and related genera, including *Alloprevotella,* have been previously associated with EC (63–65), and *Alloscardovia* have been proposed as biomarkers of human papillomavirus and cervical cancer (66). These organisms comprise a microbial fingerprint of EC, which has demonstrated potential in Chinese cohorts for non-invasive disease detection without the need for biopsy (26). That *Lautropia* spp. was the strongest predictor of health is an observation of note. A single species exists in standing nomenclature (*L. mirabilis*) (67); however, a second species has also been described, differing at the level of whole genome average nucleotide identity (68). *Lautropia* has been isolated from a variety of oral microenvironments, including saliva, dental plaque, and gingival biofilms, with a few reports considering it an opportunistic pathogen (69). Further studies are needed to understand how microbes in the upper gastrointestinal tract influence carcinogenesis in the mucosa.

While sequencing technologies are not yet widely available in the clinical context, rapid developments in inexpensive and field-deployable sequencers offer promise for diagnostics in the near future (70). Alternatively, PCR-based assays may provide utility; however, sequencing at scale may be less resource-intensive than highly multiplexed PCR for the purposes of community-level screening. When performed at scale, high-throughput DNA extraction, amplicon library preparation, and highly multiplexed sequencing using the latest generation of patterned-flow cell short-read sequencers like the Illumina NextSeq 2000/NovaSeq X could provide a first-pass screening tool at a consumables cost of <$10/sample.

There are several limitations to be considered with respect to the interpretation of the findings. Given that our study was not prospective, we are unable to establish causality of shifts in microbial community structure in the development of EC, and we are not able to validate our approach to predict future cancer development. While this has been previously reported in American populations (25), data were not made available for direct comparison; however, through qualitative analysis, many key indicator organisms overlap between our report and the American cohort, including reductions in commensal *Neisseria* spp. Further mechanistic studies are required to understand the extent to which oral microbes are a driver, or a marker, of esophageal carcinogenesis. This study design may also detect reverse causality, in which the altered environment of the oral cavity, relating directly or behaviorally to esophageal cancer, drives the shift in microbiota composition; however, this does not preclude the use of oral microbiota as a screening tool (71). Controls were drawn from individuals accompanying EC patients to clinical sites, which may have biased the demographics of the healthy control group. By the nature of the recruitment strategy and EC’s occurrence patterns, the EC group was skewed toward older males. This was accounted for in statistical models; however, future studies should focus on prospective studies with age and sex-matched controls. Comparison of microbiota sequencing data between studies is imperfect owing to many technical variables, which may be collectively termed “study effects” and explain the greatest proportion of variance in such analyses (48). This includes important variables such as sample preservation, although the use of 95% ethanol for preservation at room temperature has been previously validated (72). Differences in sample extraction, library preparation, and sequencing can further render challenges in the direct comparison of data sets (44). Despite these challenges, both qualitative and quantitative comparisons between data sets reveal similarities in composition and ESCC-associated microbiota, suggesting that the biological signal exceeds background technical noise. Furthermore, by the nature of data availability, populations are not matched on important covariates such as sex or age, and these data are often incomplete in public repositories. We performed functional characterization of the microbiota by inferring pathway abundances (73) rather than through direct metagenomic sequencing due to low sample biomass and high host DNA content in saliva, rendering metagenomic sequencing resource-prohibitive. As such, prediction of specific clade variable traits, including those related to virulence, may be imprecise (74). Furthermore, careful interpretation is required when using machine learning approaches to avoid overfitting. This was mitigated through a careful internal and external validation strategy, minimizing the potential for overfitting and data leakage; however, additional international cohorts and data sets would be needed for conclusive validation and model refinement.

In conclusion, the salivary microbiota of rural Ethiopians is highly diverse with an apparent bifurcation into two subtypes defined by differing diversity, microbial composition, function, and absolute abundance. While sex and alcohol use are significant determinants of oral microbiota composition, the factors that determine the community subtype remain to be determined but cannot be explained by technical variables. The lower diversity and abundance cluster 2 was over-represented in individuals with esophageal cancer, potentially indicating a protective role of the oral microbiota against esophageal cancer; however, this signature also demonstrated power as a non-invasive diagnostic tool that could also predict current disease state in a Chinese cohort. Understanding the mechanisms that drive these associations and their directionality will be key for the early detection and prevention of this disease, in particular, esophageal squamous cell carcinoma, which disproportionately impacts individuals in East Africa and Central Asia.

## MATERIALS AND METHODS

### Participant recruitment and sample collection

Participants were recruited from the Oromia catchment area of the Adama Hospital Medical College, Adama General Hospital and Medical College, Muse General Hospital, Asella Rehoboth Hospital, and Meda Wolabu Hospital. The enrollment criteria were either being newly diagnosed with EC and being treatment-naive or being a healthy control with no significant co-morbidities of diabetes mellitus, HIV infection, other cancers, and/or hypertension. Healthy controls were drawn from individuals who accompanied EC participants to the recruiting medical centers. Healthy controls were not matched on covariates, including age or sex, and were not necessarily co-habilitating relatives. Analysis of Bray-Curtis dissimilarities (see “Microbiota characterization” below) between EC participants and their accompanying controls (*N* = 65 pairs) did not reveal significant differences in intra-pair vs. inter-pair dissimilarity, providing no evidence of potential confounding by study pair (*P* = 0.418, Welch’s *t*-test). Due to sample availability, 38% of the participants did not have a paired control/EC participant. As such, data from this study were treated as a cross-sectional design. Individuals receiving antibiotics in the past 2 weeks and those with active oral disease were excluded from the study. Active oral disease was defined as uncontrolled periodontitis, active oral mucosal infection requiring systemic therapy, and/or acute dental abscess. After obtaining written informed consent, participants were interviewed using a semi-structured questionnaire to obtain socio-demographic and behavioral data relevant to EC risk factors. Information on tobacco usage (both smoking and chewing), alcohol use, and khat use was collected as binary outcomes based on current self-reported usage. Tumor histological types were identified by hematoxylin and eosin-stained microscopic histological examination. Samples were collected between January and July 2022. Sample size was determined based on sample availability from the larger cohort. A formal power calculation was not performed.

Saliva samples were collected between 8:30 and 11:00 a.m. in the morning in a fasted state before the participants had consumed a meal. Data on other metrics of oral health, including the number of teeth and cavities, were not collected. For microbiota analysis, participants were instructed to deposit approximately 5 mL of saliva into a sterile 25 mL conical tube. The saliva was then mixed with an equal volume of 96% ethanol before transfer for long-term storage. For mycotoxin exposure, a whole blood sample of 5 mL was collected using an EDTA-coated tube from each participant. Plasma was separated by centrifugation at 5,000 rpm for 5 min, transferred to a sterile cryotube using a sterile pipette. Both saliva and plasma were stored at −80°C until processing. Samples were shipped on dry ice to PSU. Total time between sample collection and processing was up to 1 year.

### Microbiota characterization

We characterized saliva samples from 211 individuals from the 322-participant cohort previously described on the basis of sample availability and viability (45). Samples were processed in the One Health Microbiome Co-laboratory at Penn State; 200 µL of each saliva sample was extracted using the ZymoBiomics 96 MagBead kit (Zymo D4308), following the manufacturer’s protocol. Mechanical disruption was performed using a FastPrep96 (MPBio), with a total disruption time of 5 min. Positive controls consisting of a defined community containing eight bacteria were included, with a reported purity of <0.01% contaminant DNA (Zymo D6300). Ten negative controls consisting of only extraction reagents were included across plates. gDNA subsequently underwent primary amplification of the V4 16S rRNA region using the following primers: V4_515Fmod_Nextera, TCGTCGGCAGCGTCAGATGTGTATAAGAGACAG*GTGYCAGCMGCCGCGGTAA*, and V4_806Rmod_Nextera, GTCTCGTGGGCTCGGAGATGTGTATAAGAGACAG*GGACTACNVGGGTWTCTAAT*, wherein the italicized portion represents the amplification primer and the remainder is used for subsequent indexing and sequencing. Samples were amplified using a high-fidelity enzyme (KAPA HiFi Hot Start PCR KK2502) in the presence of SYBR green (Sigma S9430) for 5 min at 95°C, followed by 32 cycles of 98°C for 20 s, 55°C for 15 s, and 72°C for 60 s. Reactions were monitored in real time using a QIAquant 384 (Qiagen). Lack of amplification of negative controls was further confirmed through gel electrophoresis to ensure the absence of a true amplicon. All samples, including negative controls, were subsequently diluted 100× and indexed using 10 cycles of the same reaction conditions with 1 µM unique dual index (UDI) sequences available at github.com/BisanzLab/OHMC_Colaboratory. Amplicons were subsequently quantified using Quant-it Picogreen (Life Technologies P7589) and pooled at equimolar concentrations. Negative controls, despite failed reactions with no detectable product, were pooled at the full available volume (9 µL). All liquid handling steps were performed using an Integra mini96 or an Opentrons OT-2. The pooled library was subsequently size-selected using both gel (Qiagen MinElute 28604) and magnetic capture beads (Ampure XP Beckman A63881). The final library was inspected by Tapestation 4200 (D1000) and quantified using the NebNext library quantification kit (NEB E7630S). The library was loaded on a P1 600 cycle kit with 40% PhiX using an Illumina NextSeq 2000 sequencing at 270 × 270. Raw sequencing data are available in the NCBI Sequence Read Archive (SRA) under accession PRJNA1137386.

The resulting data were processed using v2.1 of the following script: github.com/BisanzLab/OHMC_Colaboratory/blob/main/analysis_scripts/AmpliconSeq_q2.Rmd. Briefly, the reads were processed using QIIME2 version 2023.5. Primer sequences were removed, allowing for an error rate of 0.15, discarding any read without a valid primer sequence on both ends. Next, reads were denoised and overlapped using Dada2 with chimeras removed using the default consensus method. Taxonomy was assigned using the Dada2 taxonomic classifier against the SILVA version 138.1 database. Metagenomic inference was performed from subsampled ASV data using PICRUSt v2.4.1 using the MetaCyc database.

A total of 211 oral samples were sequenced, resulting in a mean post-processing read depth of 129,651 ± 92,473 reads (mean ± SD), with a median depth of 115,188 (10,992–556,826) (median [range]). There was no significant difference between EC (*N* = 103) and healthy controls (*N* = 108) in terms of read depth (*P* = 0.94, Welch’s *t*-test, 130,137 ± 83,934 healthy vs. 129,141 ± 101,068 EC participants). Samples were randomized across 4 × 96-well plates for DNA extraction, with library preparation performed in a single 384-well plate to minimize potential batch effects in processing. All extraction and library preparation reagents were obtained from single bulk-purchased lots and were performed over the period of sequential days. No significant effects on alpha or beta diversity were observed across extraction plates, indicating no detectable batch effects. As such, no further batch effect removal algorithms were applied. An analysis of potential batch effect is included in Fig. S5, with further analyses presented in this manuscript’s associated GitHub repository. A total of 10 negative controls (DNA extraction with reagents only) were included across the four plates. While successful amplification by qPCR or gel electrophoresis was not observed in the negative controls, they were still pooled into the sequencing run at the maximum available reaction volume. Negative controls resulted in <1,592 reads per sample (771 ± 393). Only two features could be reproducibly observed across negative controls: Burkholderiales and *Escherichia coli*, known reagent contaminants (75), which are also present in the oral microbiota. A total of four positive controls of a defined community were included (134,136 ± 73,325 reads; see Materials and Methods). Fewer than 43 reads could be assigned to organisms not reported in the reference community standard (0.0097% ± 0.0087%, range 0%–0.02%; 17.3 ± 18.3 reads, range 0–43 reads), an approximation of the manufacturer’s reported purity of < 0.01% foreign microbial DNA. A summary of potential contaminants and their abundances in technical controls and oral samples is available in Table S1. All analyses were replicated after conservative filtering of potential contaminant taxa to ensure that major findings were not driven by potential contaminant taxa; however, given that the origin of low-abundance features is difficult to determine and may be driven by both biological and technical sources, including index hopping, all taxa were retained for the downstream analysis presented in this manuscript.

### Microbiota analysis

All data were processed using R 4.5.0 QIIME 2 artifacts were imported using qiime2R. Samples with less than 10,000 reads were discarded prior to downstream processing, which included all negative controls. Alpha diversity metrics were generated after subsampling to the lowest available read depth for the sample set of interest using vegan::diversity (v2.6-6.1) for Shannon’s diversity index and picante::pd (v1.8.2) for Faith’s phylogenetic diversity and ASV (species) richness. The lowest subsampling depth was 10,992 reads. CLR Euclidean (Aitchison) distances were calculated on genus-summarized abundances without subsampling by first using qiime2R::make_clr to conduct a centered-log2-ratio transformation, followed by stats::dist to calculate the Euclidean distance. Clustering was performed with the partitioning around medoids method using cluster::pam (v2.1.6) and CLR Euclidean (Aitchison distance) with the gap statistic calculated using cluster::clusGap. The number of clusters was determined based on selecting the smallest number of clusters *k* such that the gap (*k*) ≥ gap (*k* + 1) − SE (*k* + 1).

### Absolute quantification of saliva microbial load

Total salivary bacteria were quantified by qPCR analysis targeting the 16S rRNA gene. The microbial DNA was extracted with the ZymoBiomics 96 MagBead kit (Zymo D4308) as mentioned above. The assay utilized the 891F forward primer: TGGAGCATGTGGTTTAATTCGA, 1003R reverse primer: TGCGGGACTTAACCCAACA, and 1002P probe: [6FAM]CACGAGCTGACGACARCCATGCA [BHQ1], each at 200 nM. The qPCRs were amplified using iTaq Universal Probes Supermix (BioRad 1725132) on a BioRad CFX384 thermocycler following the procedure: initial denaturation at 95°C for 5 min, and 40 cycles of denaturation at 95°C for 5 s, and annealing/extension at 60°C for 15 s. Absolute quantification was performed using a standard curve of purified 8F/1542R primer amplified *E. coli* MG1655 DNA (76). Each assay was performed in triplicate in a 10 µL reaction mixture.

### Statistical analysis

Univariate analysis was performed using appropriate base R functions for Welch’s *t*-test (stats::t.test), Mann-Whitney *U* test (stats::wilcox.test), or Fisher’s exact test (stats::fisher.test) as indicated in figure legends. Statistical analysis of distances was performed by PERMANOVA (vegan::adonis2). Where relevant (Fig. 4D), confounding variables were included in the adonis2 model as distance ~ cancer status + age + sex + alcohol consumption. Statistical analysis of differentially abundant taxon features was performed using ALDEx2 (v1.36.0) with centered log2 ratio normalization using either Welch’s *t*-test for cross-sectional analysis of community type (ALDEx2::aldex) or generalized linear model accounting for confounding variables (ALDEx2::glm), both using Benjamini-Hochberg false discovery rate correction. In the ALDEx2 GLM, the formula was feature abundance ~ cancer status + sex + age + alcohol consumption. GLMs for alpha diversity metrics were performed using stats::glm. Model terms were subsequently reduced to minimize the Akaike information criterion (AIC). Differential pathway abundances were performed by normalizing to relative abundance and conducting a log2-transformation after the addition of a pseudocount corresponding to two-thirds of the lowest per-feature abundance. Analysis was subsequently performed by Welch’s *t*-test with Benjamini-Hochberg false discovery rate correction. Odds ratios (OR) were extracted from a Fisher’s exact test or by multivariate logistic regression. In the multivariate logistic regression, disease status was encoded as a binary outcome, and a GLM with a binomial distribution and logit link was fit with disease status as the dependent variable and cluster as the primary independent variable, adjusting for age, sex, and alcohol consumption. Model coefficients were exponentiated to obtain ORs. This analysis was performed using the stats::glm() function, with the results summarized using the broom package. All plots were generated using ggplot2 and relevant extensions. Error bars represent standard error (SE) unless otherwise noted.

### Comparison to global oral microbiota data

Ethiopian data sets generated in this study were compared to 16S rRNA gene V4 raw data sets, including those from Tanzania, Venezuela, Uganda, and westernized populations represented in the American Gut Project as described by Ademola-Popoola et al. (77). Quality filtering of the data was performed with the “q2-quality-filter” plugin, and denoising was carried out using Deblur (78) via the “q2-deblur” plugin, with a trim length of 150 bases, resulting in amplicon sequence variants (ASVs). The representative sequences were aligned using MAFFT (79) with the “q2-alignment” plugin, and a phylogenetic tree was constructed using FastTree2 (80) via the “q2-phylogeny” plugin. The data set was then filtered to include only adults, oral samples, single-timestamp samples, and those without antibiotic use or other treatments that could alter oral microbiome composition using the “qiime feature-table filter-samples” plugin. To ensure data robustness, features present in only one sample and with < 5 occurrences across all samples were removed. Decontamination was applied using all available project controls (i.e., extraction reagent controls and no-template amplification controls) using decontam, and sequences associated with chloroplasts and mitochondria were removed. Finally, samples with fewer than five taxa and high levels (>40%) of identified contaminants were excluded, including individual “11492.A19489.TW99” (46.592% contamination from Synechococcus) and individual “11492.A19451.TW51” (45.833% contamination from JG30-KF-CM45). Additionally, duplicate samples were also removed (i.e., “11492.A19451.KG59e”). Alpha and beta diversity analyses were performed as described above. Statistical analysis of Shannon’s diversity was performed by ANOVA with Tukey HSD *post hoc* test. Accessions and code to obtain these data sets are available at https://github.com/Iyunoluwa-J/Ethiopian_oral_cancer_microbiome.

### Model training and validation on the external cohort

A literature search was conducted to identify additional oral microbiota studies of health and EC individuals, which had used Illumina-based short-read amplicon sequencing covering the V4 region for characterization. Two Chinese studies were identified, Chen et al. (Bioproject accession PRJNA964904) and Jiang et al. (PRJNA853196), to have available sequencing data and unambiguous metadata. Both data sets were V3-V4 sequencing performed on an Illumina NovaSeq with 150 × 150 reads. Due to issues with denoising and overlapping V3-V4 amplicons on this platform, only the reverse read covering the V4 region was carried forward for denoising, taxonomic assignment, and diversity characterization as above. For cross-cohort comparisons, a Monte-Carlo cross-validation strategy was used wherein 100 iterations of the following training/testing strategy were applied. First, the Ethiopian cohort was randomly divided into a training set (139 participants) and a test set (72 participants). A random forest classifier (randomForest v4.7-1.1) model predicting EC vs. control was trained on genus-summarized proportional abundances from the training set and then used to predict both the test sets and the external cohorts. ROCs and AUROCs were derived using the ROCR (v1.0-11) and pROC (v1.18.5) packages. Feature importances were derived by extracting the per-feature mean decrease in GINI coefficients from each iteration of the classifier. AUROCs and GINI coefficients were reported using the distribution of each iteration.

### Multiple mycotoxin biomonitoring

Mycotoxin determination in plasma samples is described in Mulisa et al. (45). Briefly, 300 µL of acetonitrile was added to 300 µL of each plasma sample for protein precipitation. Samples were vortexed for 2 min and centrifuged at 3,300 × *g* for 15 min at 4°C using a Multifuge 3 S-R centrifuge from Heraeus (Hanau, Germany). The supernatants were transferred to Eppendorf glass tubes in a Turbo Vap LV evaporator from Biotage (Dusseldorf, Germany) and evaporated at 40°C under gentle nitrogen gas flow until completely dry. The residues were re-dissolved in 150 µL of injection solvent (60:40 [vol/vol] mobile phase A/mobile phase B) by vortexing for 2 min and then centrifuged at 2,100 × *g* for 1 min. The dissolved residue was transferred to a tube with a PVDF centrifuge filter of 0.22 µm from Millipore (Cork, Ireland) and centrifuged at 9,000 × *g* for 5 min at 4°C. The filtrate was transferred into a HPLC vial and analyzed by liquid chromatography-tandem mass spectrometry (LC-MS/MS) using a Water Acquity UPLC I-class system coupled to triple quadrupole XEVO TQ-XS mass spectrometer equipped with an electrospray ionization source. Chromatographic separation was done in an Acquity UPLC HSS T3 column (1.8 µM particle size, 2.1 mm id × 100.0 mm) with a matching VanGuard precolumn (5 mm). Analytes were quantified against authentic analytical standards of 39 mycotoxins (3-acetyldeoxynivalenol, aflatoxin B1 [AFB1], aflatoxin B2 [AFB2], aflatoxin G1, aflatoxin G2, aflatoxin M1, alternariol methylether, alternariol, beauvericin, citrinin [CIT], cyclopiazonic acid [CPA], diacetoxyscirpenol, deepoxy-deoxynivalenol, deoxynivalenol [DON], enniatin A, enniatin A1, enniatin B [ENN B], enniatin B1, fumonisin B1 [FB1], fumonisin B2, fumonisin B3, fusarenone-X, hydrolyzed fumonisin B1, HT-2 toxin [HT2], neosolaniol, nivalenol [NIV], ochratoxin A [OTA], ochratoxin alpha, roquefortine-C, sterigmatocystin, tenuazonic acid [TA], T-2-toxin [T2], T-2 tetraol, zearalanone [ZAN], alpha-zearalanol, beta-zearalanol, zearalenone, alpha-zearalenol [α-ZEL], and beta-zearalenol) using matrix-matched calibration curves and normalized using an appropriate isotopically labeled internal standard (^13^C-AFB1, ^13^C-CIT, ^13^C-DON, ^13^C-FB1, ^13^C-HT2, ^13^C-T2, and ^13^C-TA).

## Data Availability

Sequencing data are available via the NCBI Sequence Read Archive under BioProject PRJNA1137386. Processed data and code to replicate analyses are available at https://github.com/BisanzLab/EOSC_manuscript. Requests for further information and resources should be directed to and will be fulfilled by the corresponding author. A STORMS checklist is available at figshare at https://doi.org/10.6084/m9.figshare.30223753.

## SUPPLEMENTAL MATERIAL

Supplemental Figures msystems.00232-26-s0001.pdf Figures S1 to S5.

Supplemental Tables msystems.00232-26-s0002.xlsx Tables S1 to S5.

ASM does not own the copyrights to Supplemental Material that may be linked to, or accessed through, an article. The authors have granted ASM a non-exclusive, world-wide license to publish the Supplemental Material files. Please contact the corresponding author directly for reuse.
