# Supplementary material for: Multi-cohort comparative analysis of salivary microbiotas reveals rural Ethiopians harbor a distinct composition correlated with lower esophageal cancer prevalence
Source: mSystems. 2026 Jun 2;11(6):e00232-26. doi: 10.1128/msystems.00232-26 (PMC13288984; doi:10.1128/msystems.00232-26)
Supplement: Supplemental Figures — Figures S1 to S5. [file msystems.00232-26-s0001.pdf]

Multi-cohort comparative analysis of salivary microbiotas reveals rural Ethiopians harbor a distinct composition correlated with lower esophageal cancer prevalence

Girma Mulisa[a,b], Jingcheng Zhao[c], Geda Lelissa[d], Iyunoluwa J. Ademola-Popoola[e,f,g], Anastacia Marie Diaz[c], Laura S. Weyrich[e,g,h], Adane Mihret[a,i], Tufa Gemechu[j], Abate Bane[k], Roger Pero-Gascon[l], Marthe De Boevre[l], Sarah De Saeger[l,m], Tamrat Abebe[a,n], and Jordan E. Bisanz[c,f,n,#]

[a]Department of Microbiology, Immunology and Parasitology, Addis Ababa University, Ethiopia
[b]Department of Biomedical Sciences, Adama Hospital Medical College, Adama, Ethiopia
[c]Department of Biochemistry and Molecular Biology, Pennsylvania State University, University Park, PA, USA
[d]Department of Internal Medicine, St. Paul's Hospital Millennium Medical College, Addis Ababa, Ethiopia
[e]Department of Anthropology, Pennsylvania State University, University Park, PA, USA
[f]One Health Microbiome Center, Huck Life Sciences Institute, University Park, PA 16802, USA
[g]Rock Ethics Institute, Department of Bioethics, Pennsylvania State University, University Park, PA 16802, USA
[h]School of Biological Sciences, University of Adelaide, Adelaide, South Australia, Australia
[i]Armauer Hansen Research Institute, Ministry of Health, Addis Ababa, Ethiopia
[j]Department of Pathology, School of Medicine College of Health Sciences, Addis Ababa University, Ethiopia
[k]Department of Internal Medicine, School of Medicine College of Health Sciences, Addis Ababa University, Ethiopia
[l]Center of Excellence in Mycotoxicology and Public Health, Faculty of Pharmaceutical Sciences, Ghent University, Belgium
[m]Department of Biotechnology and Food Technology, Faculty of Sciences, University of Johannesburg, South Africa
[n]Senior author

Address correspondence to Jordan E. Bisanz, jordan.bisanz@psu.edu

Additional File 1. Figures S1-S5.

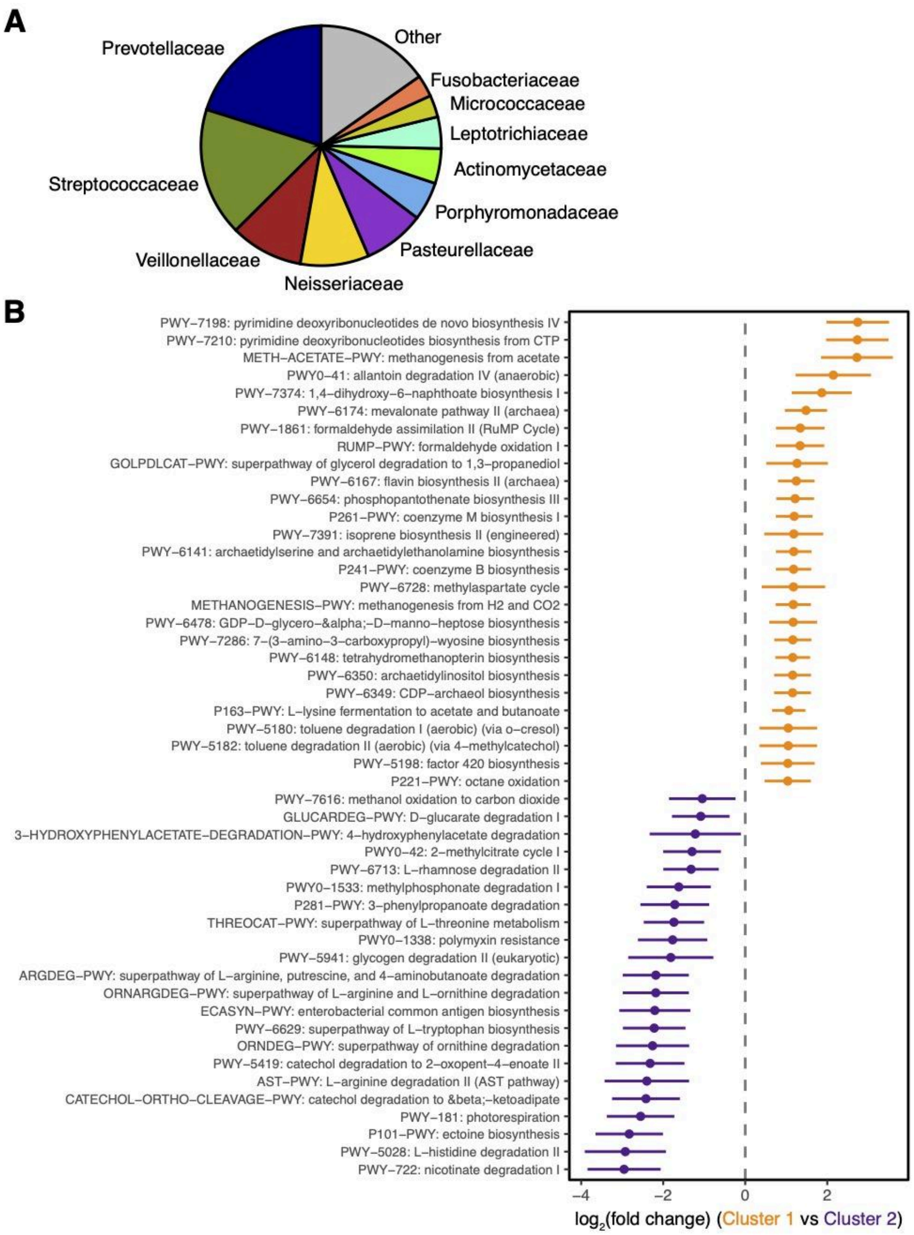

Figure S1. Characterization of oral microbiota in healthy cohort. (A) Average family-level microbiome composition of healthy individuals (N=108). Averages were computed as the compositional (Aitchison) center. **(B)** Healthy community clusters are inferred to be functionally distinct. Metagenomic inference (PICRUSt, *see Methods)* uncovers an enrichment in amino acid metabolism in cluster 2 while numerous pathways associated with archaea are enriched in community cluster 2. N=108 healthy participants, $N_{Cluster1}$=64, $N_{Cluster2}$=44; FDR-corrected Welch's t-test of log2-normalized pathway abundances. Error bars represent 95% confidence intervals. Significance was determined as FDR<0.05 and an absolute fold change >1.

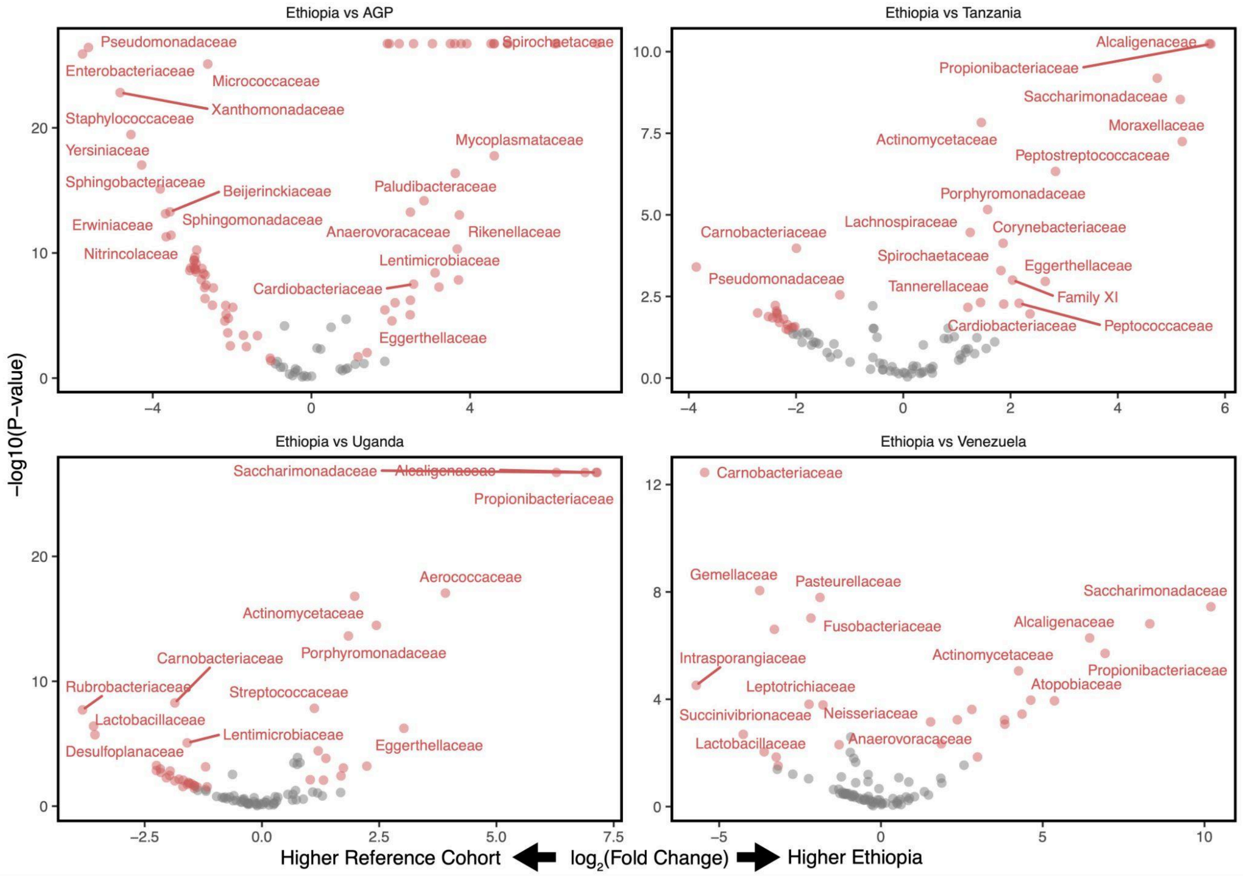

Figure S2. Differentially abundant families between healthy Ethiopians and international cohorts. Volcano plots show families with significance determined as absolute log2(fold change)>1 and FDR<0.1 determined by ALDEx2. N_{AGP}=492, $N_{Ethiopia}$=107, $N_{Tanzania}$=36, N_{Uganda}=97, $N_{Venezuela}$=16.

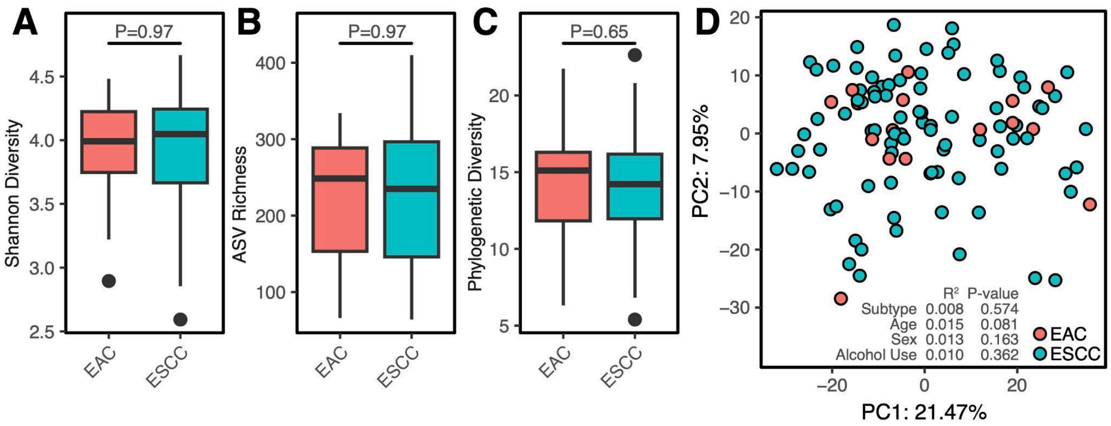

Figure S3. There is no significant difference in microbiota composition between esophageal cancer subtypes ESCC and EAC. Microbial diversity is not significantly reduced irrespective of choice of alpha diversity metric including **(A)** Shannon's diversity index, **(B)** ASV richness, and **(C)** phylogenetic diversity. **(D)** Visualization by PCoA demonstrates no significant variation in microbiome composition by cancer subtype which is supported by statistical analysis (inset, PERMANOVA). Statistical analysis for planes A and C by GLM with covariates of sex, age, alcohol use. Statistical analysis for panel B same with negative binomial GLM. N_{ESCC}=87, N_{EAC}=16 for all panels.

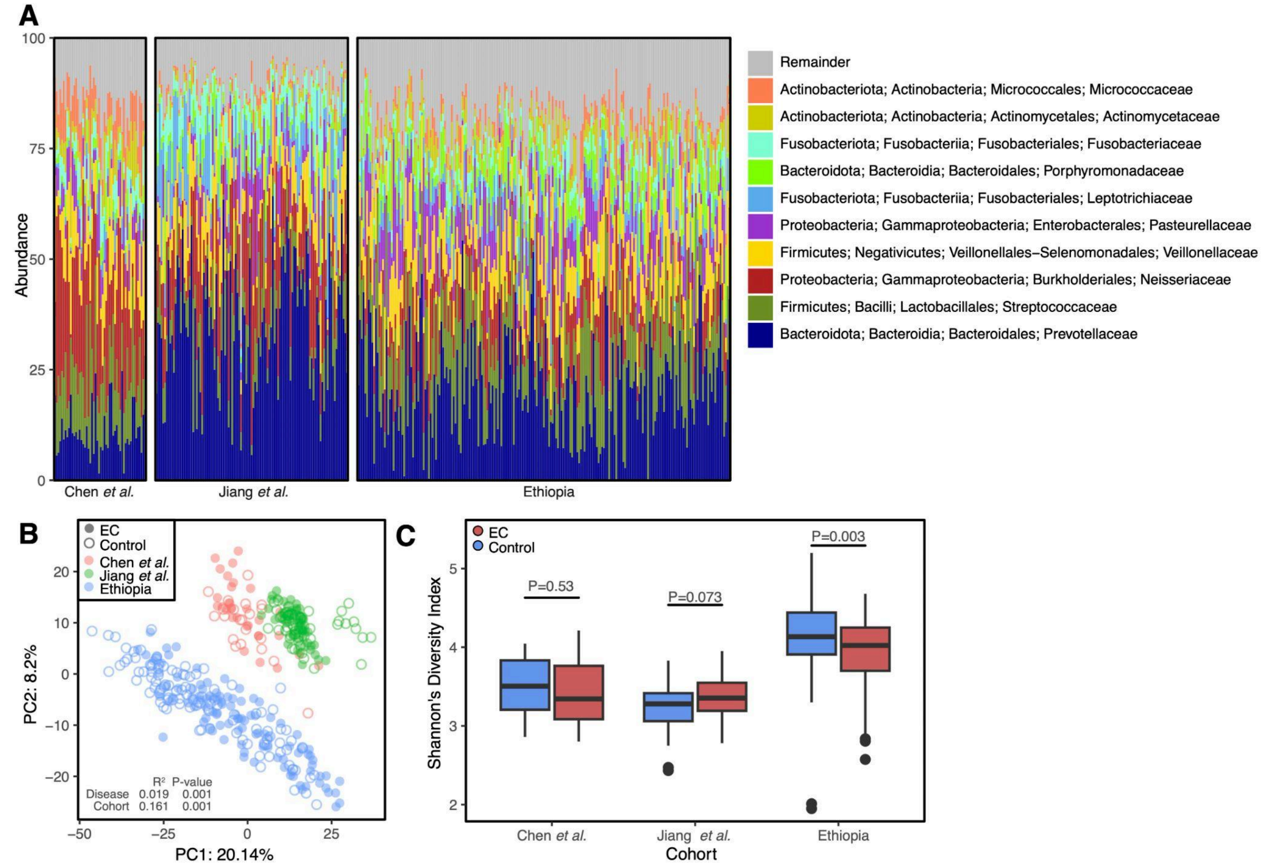

Figure S4. Esophageal cancer cohorts are unique in composition and diversity. (A) Taxonomic bar plots summarized at the family level demonstrate similar presence of the major families in the oral microbiota; however, distinct differences in the abundance of these families includes a lower abundance of Prevotellaceae and an increase in Neisseriaceae in the Chen *et al.* cohort. **(B)** PCoA of CLR Euclidean distances demonstrates that cohorts are compositionally distinct explaining the major source of variation, while there is still a significant effect of cancer status (PERMANOVA table inset). **(C)** Chinese cohorts do not display differential alpha diversity between case and controls (Welch's t-test). In all panels: $N_{Ethiopia}$=211 (N_{cases}=103, $N_{controls}$=108), N_{Chen}=52 (N_{cases}=31, $N_{controls}$=21), N_{Jiang}=109 (N_{cases}=56, $N_{controls}$=53).

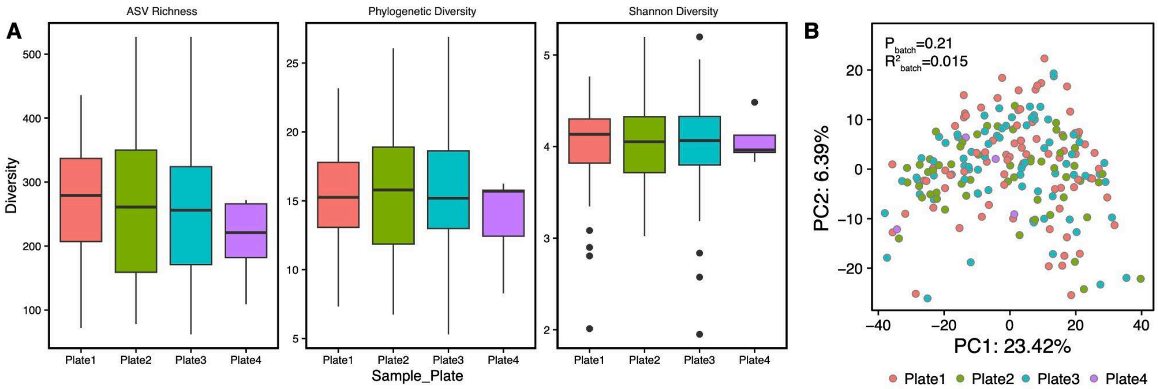

Figure S5. Comparison of (**A**) alpha and **(B)** beta diversity by extraction plate. No statistically significant effects were observed (panel A ANOVA, panel B PERMANOVA).
